# *Ex Vivo* Raman Spectrochemical Analysis Using a Handheld Probe Demonstrates High Predictive Capability of Brain Tumour Status

**DOI:** 10.3390/bios9020049

**Published:** 2019-03-30

**Authors:** Danielle Bury, Camilo L. M. Morais, Katherine M. Ashton, Timothy P. Dawson, Francis L. Martin

**Affiliations:** 1 School of Pharmacy and Biomedical Sciences, University of Central Lancashire, Preston PR1 2HE, UK; deb11@doctors.org.uk (D.B.); CDLMedeiros-de-morai@uclan.ac.uk (C.L.M.M.); 2 Neuropathology, Royal Preston Hospital, Lancashire Teaching Hospitals NHS Trust, Sharoe Green Lane, Preston PR2 9HT, UK; Katherine.ashton@lthtr.nhs.uk (K.M.A.); timothy.dawson@lthtr.nhs.uk (T.P.D.)

**Keywords:** brain tumour diagnosis, classification, forward feature extraction algorithm, intraoperative use, Raman spectroscopy, Raman probe

## Abstract

With brain tumour incidence increasing, there is an urgent need for better diagnostic tools. Intraoperatively, brain tumours are diagnosed using a smear preparation reported by a neuropathologist. These have many limitations, including the time taken for the specimen to reach the pathology department and for results to be communicated to the surgeon. There is also a need to assist with resection rates and identifying infiltrative tumour edges intraoperatively to improve clearance. We present a novel study using a handheld Raman probe in conjunction with gold nanoparticles, to detect primary and metastatic brain tumours from fresh brain tissue sent for intraoperative smear diagnosis. Fresh brain tissue samples sent for intraoperative smear diagnosis were tested using the handheld Raman probe after application of gold nanoparticles. Derived Raman spectra were inputted into forward feature extraction algorithms to build a predictive model for sensitivity and specificity of outcome. These results demonstrate an ability to detect primary from metastatic tumours (especially for normal and low grade lesions), in which accuracy, sensitivity and specificity were respectively equal to 98.6%, 94.4% and 99.5% for normal brain tissue; 96.1%, 92.2% and 97.0% for low grade glial tumours; 90.3%, 89.7% and 90.6% for high grade glial tumours; 94.8%, 63.9% and 97.1% for meningiomas; 95.4%, 79.2% and 98.8% for metastases; and 99.6%, 88.9% and 100% for lymphoma, based on smear samples (κ = 0.87). Similar results were observed when compared to the final formalin-fixed paraffin embedded tissue diagnosis (κ = 0.85). Overall, our results have demonstrated the ability of Raman spectroscopy to match results provided by intraoperative smear diagnosis and raise the possibility of use intraoperatively to aid surgeons by providing faster diagnosis. Moving this technology into theatre will allow it to develop further and thus reach its potential in the clinical arena.

## 1. Introduction

Brain tumours account for 3% of all tumours diagnosed annually [1]. Whilst this comprises a small proportion of total cancer burden, the difficulty of complete removal of the tumour is inherent. High-grade tumours can be infiltrative and when operating within the brain the risk of removing crucial structures in a bid to free the patient of the tumour, yet risk leaving them with significant neural deficit is ever present. Up to 75% of tumour resections are thought to leave behind viable tumour, though there is a survival benefit to improved/complete resection [2,3]. Therefore, any new technique available to highlight residual tumour, thus improving outcome and resection, yet reducing the non-tumour tissue removed would be beneficial. Currently, the use of 5-aminolevulinic acid (5-ALA) does allow for fluorescence of tumour cells in order to aid resection; however, this is imperfect. It can be difficult to tell apart tumour from background fluorescence [4].

In recent years many studies have been performed using vibrational spectroscopy in an effort to improve and decrease time to cancer diagnosis and aid resection of tumours. Vibrational spectroscopy includes two complementary techniques: Raman and attenuated total reflection Fourier-transform infrared (ATR-FTIR) spectroscopy. Raman spectroscopy detects chemical bonds via scattering of photons due to bond vibrations, whereas ATR-FTIR spectroscopy measures energy absorbance after excitation by an IR beam following reflection of the beam via an internal element (usually crystal) [5]. Both generate a ‘fingerprint’ of the elements within the examined sample, which can be examined to determine differences between them [5]. The majority of these studies have been ex vivo, with a move in recent years to increase the number of in situ studies [6], though these have yet to demonstrate definitive results. The need to test fresh tissue is crucial, to overcome any spectral changes seen due to formalin fixation or freezing artefact [7,8]. The use of gold nanoparticles in conjunction with Raman spectroscopy, known as surface enhanced Raman scattering (SERS) has previously been shown to improve the Raman signal received, reducing signal-to-noise ratio (SNR) and thus enhance the spectral quality [9]. This method uses molecules adsorbed onto the target surface prior to spectral acquisition, it has previously shown promise when used particularly with blood products to detect cancer [10].

The ability to use a probe intraoperatively, for example in brain surgery, and tell the surgeon in real-time if the tissue is cancerous or not would be greatly beneficial and perhaps the most useful area for Raman spectroscopy to make its clinical entry. Many areas within the cancer care pathway have been considered for targeting by spectroscopy, yet this is likely to be the best target location [10,11]. Stables et al. proposed a sound method to enable the surgeon to detect differences in the brain tissue found using spectroscopy as a method to provide real-time feedback [12]. This is an interesting suggestion, and certainly there is a need to develop technology to provide the surgeon with an answer without the need to interpret spectra. Desroches et al. used a handheld Raman probe intraoperatively with an accuracy of 87% to determine brain tumour from non-tumour tissue [13]. They then followed the study with the development of an optical biopsy needle for use during brain tumour biopsies. Following validation in an animal model, they tested their system during human brain surgery with an accuracy of 84%, sensitivity of 80% and specificity of 90% for tumour detection. These results were from comparing Raman spectra to biopsy results where the majority of the biopsy comprised tumour tissue [14]. This shows exciting potential; however, due to light contamination much of their study required procedures to be performed in darkness. Other types of Raman probes, such as based on stimulated Raman scattering, have been used in many biological applications, in particular for intracellular sensing and imaging [15].

Handheld probes have also been used for lymph node, breast and cervical testing. Horsnell et al. demonstrated that a handheld Raman probe used to determine the presence of cancer within sentinel lymph nodes with suspected breast cancer metastasis. They achieved sensitivities and specificities of up to 92% and 100% respectively, using frozen tissue [16]. They then went on to test lymph nodes using Raman micro-spectroscopy, and achieved concordance with histopathology in up to 91% of cases, improving as more points were assessed [17]. Within breast pathology, Haka et al. demonstrated a 93% accuracy in determining normal breast from benign or cancerous lesions [18]. They also demonstrated the potential of using Raman spectroscopy for intraoperative assessment of mastectomy margins with positive results, and possibly may have improved intraoperative results had spectroscopy been used in real-time [19]. As Raman spectroscopy is unaffected by aqueous materials therefore it is felt by the authors to be most suited to examining fresh brain tissue.

This study has been designed in order to determine the potential of the use of intraoperative SERS for brain tumour diagnosis within the neuropathology department. Raman spectral analysis of fresh brain tissue sent for intraoperative smear diagnosis was performed. The objective was to compare SERS results to both the intraoperative smear result and final formalin-fixed paraffin embedded tissue (FFPE) result. This was done to understand if SERS can aid the clinical pathway and provide results similar to conventional neuropathology, with the aim of replacing the need for an intraoperative smear diagnosis, allowing the surgeon to test tissue intraoperatively to guide diagnosis and resection in the future.

## 2. Materials and Methods

**Figure 1:**
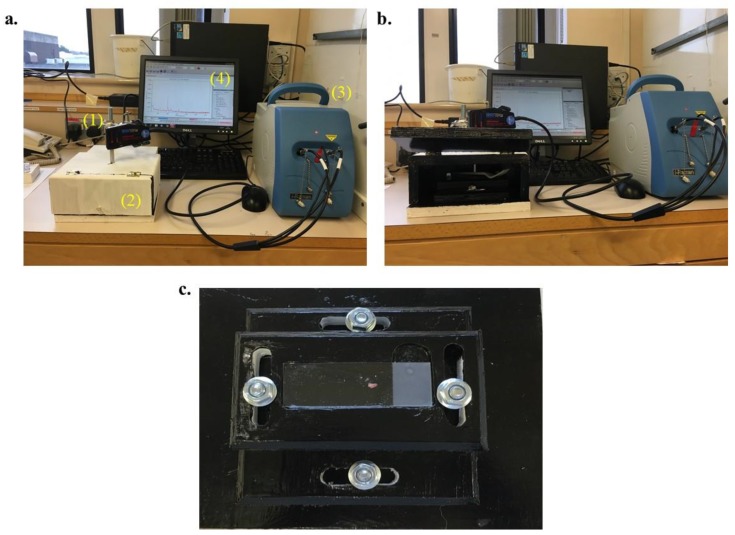
The handheld Raman probe in situ in the Neuropathology Department at Royal Preston Hospital. (**a**) Instrument setup showing (**1**) Raman probe, (**2**) sample compartment, (**3**) Raman detector and laser source, and (**4**) computer module with appropriate software. (**b**) A different view of the instrument setup with the sample compartment opened. (**c**) Sample holder with an example slide inside the sample compartment.

Prior to using the handheld Raman machine, a custom-built box (sample compartment) was required to ensure darkness when analysing the tissues. As this was being placed into a working laboratory, it would not be possible to work in darkness and it would also need to fit into a category 2 fume hood for work with fresh tissue. With this is mind, a box was custom engineered using plywood. A stage was built within this box to allow the slide to be moved in the x and y planes with a custom cut out area for the slide to be held securely. This was to allow the tissue to be accurately positioned under the probe. A clamp was then secured to the box to allow the probe to be moved in the z plane to allow it to be positioned at the correct height above the tissue. Thus allowing movement similar to a conventional light microscope. The box was painted with black paint on the inside to minimise reflection of any light entering it. It also enabled it to be wiped clean if required. This was designed to be a prototype hence the materials involved (Figure 1).

Integrating such studies in a typical clinical setting is a major challenge, especially during such serious and complicated procedures. Fresh brain tissue samples sent to the laboratory for intraoperative smear preparations were tested over a 6-month period (Table 1). Ethical approval was obtained from the BTNW brain bank (NRES14/EE/1270). We obtained *n* = 29 samples (a decent cohort size), which were analysed using an i-Raman portable Raman system with BAC100/BAC102 lab-grade Raman probe from B&W Tek from Pacer International, with software version 4.1. All consented samples arriving in the laboratory were tested over a six-month time period, typical of a routine clinical setting.

The samples tested using the Raman spectrometer were obtained from tissue sent for intraoperative smear diagnosis. This tissue was then formalin fixed along with any remaining tissue for formal neuropathological examination. The sample arrived via air-tube from theatre within 5–10 min of removal from the patient. Prior to sample analysis, a small amount of tissue (similar in size to that used for a smear preparation) [20] was placed onto a glass slide covered with aluminium foil [21] and 100 µL of 5 µg/mL BioPureTM 20 nm gold nanoparticles diluted in PBS was dropped onto the sample and left for 2 min to absorb prior to collecting 10 spectra per sample. Gold nanoparticles were used to enhance spectral quality. Each spectrum had an acquisition time of 30 s at a laser power of 75%, field of view 0.9 mm × 0.9 mm, with a 785 nm laser. In total the spectra took 5 min to acquire and 2 min to save prior to analysis. This equates to the time taken to prepare a smear preparation prior to histopathological analysis. Once analysed tissue was formalin-fixed for final histopathological diagnosis. 

Data analysis was then conducted using MATLAB R2014b software (MathWorks Inc., Natick, MA, USA) with an IRootlab toolkit [22]. The raw spectral data were initially pre-processed by cutting the region of interest, 1800–400 cm^−1^, followed by polynomial baseline correction and vector normalisation. Thereafter, principal component analysis-linear discriminant classifier (PCA-LDC) was applied for classification of the datasets on a spectral basis. The training and validation sets were split using sub-dataset generation specification algorithm within IRootlab toolkit, where the model validation was performed using 10% of samples randomly assigned to the validation set during model construction. Due to the small number of samples, each spectrum was analysed separately for a predicted tissue pathology. PCA-LDC uses PCA as feature extraction method, where the original data is decomposed into a few number of principal components (PCs) representing the majority of the information in the original dataset. The scores on each PC are then used as input variables for linear discriminant analysis (LDA). LDA works by maximizing the between-class variance over the within-class variance in order to create a linear decision boundary between the classes that provides the optimum class segregation [23]. Patient factors, including the location of the tumour and biopsy were not considered within this study. Whilst the tumour site does influence the histopathological diagnosis, many pathologists prefer to start the diagnostic process on morphology only, blinded to demographics, history and site to prevent unconscious bias. Once a morphological diagnosis or range of differential diagnoses are formulated this can be tested against the site and demographics. Therefore, the Raman analysis is essentially being used to offer the same initial analysis as a pathologist on morphology only. Demographics, history and site are part of the secondary analysis, which as this study develops in the future could then be included within the analysis.

## 3. Results

**Figure 2:**
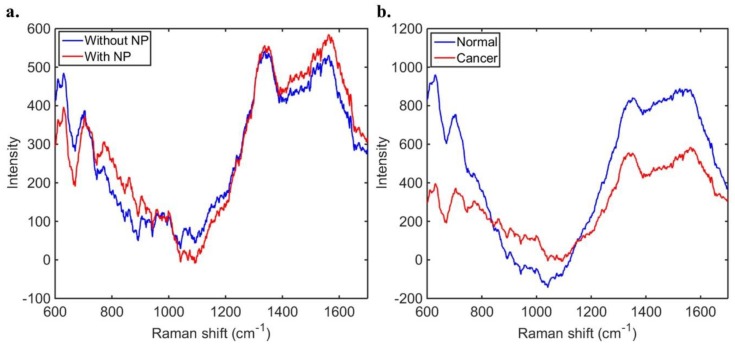
Baseline-corrected Raman spectra for (**a**) high-grade glioma tissues with and without gold nanoparticles (NP); (**b**) none tumour (normal) brain and cancer (high-grade glioma) tissues with gold nanoparticles. Each spectrum represents the average of 10 measurements in the tissue sample.

Over the 29 samples from 27 patients, 290 spectra were collected and analysed. Due to the relatively small number of samples, each spectrum was analysed separately for a predicted tissue pathology. From this, PCA-LDC was employed and receiver operating characteristic (ROC) curves generated. This was done to determine the classification accuracies of the Raman spectra as compared to both the intraoperative smear result and final FFPE histological diagnosis, followed by ROC curves to determine the accuracy of the classification model as well as its sensitivity and specificity were generated. Low-grade gliomas were considered WHO grades 1 and 2, and high-grade gliomas WHO grades 3 and 4. Meningiomas were classed as WHO grade 1. Metastatic tumours were grouped due to the range of different primary sites within the tumours tested, and as intraoperatively ‘metastasis’ is sufficient for intraoperative surgical planning. The Raman spectra with or without gold nanoparticles for the same type of sample (high-grade glioma) are shown in Figure 2a. SERS is emitted from only the molecules adsorbed on the nanoparticles surface; thus the spectrum is not always the same as the corresponding Raman spectrum without nanoparticles. In our case, although the shape of both spectra looks similar, the Raman spectrum with gold nanoparticles contains higher intensities in the regions between ~1300–1700 cm^−1^ and ~700–1000 cm^−1^. Figure 2b depicts the Raman spectra for non-tumour brain and cancer (high-grade glioma) tissue samples in presence of gold nanoparticles.

### 3.1. SERS Results Compared to Intraoperative Smear Preparation

**Figure 3:**
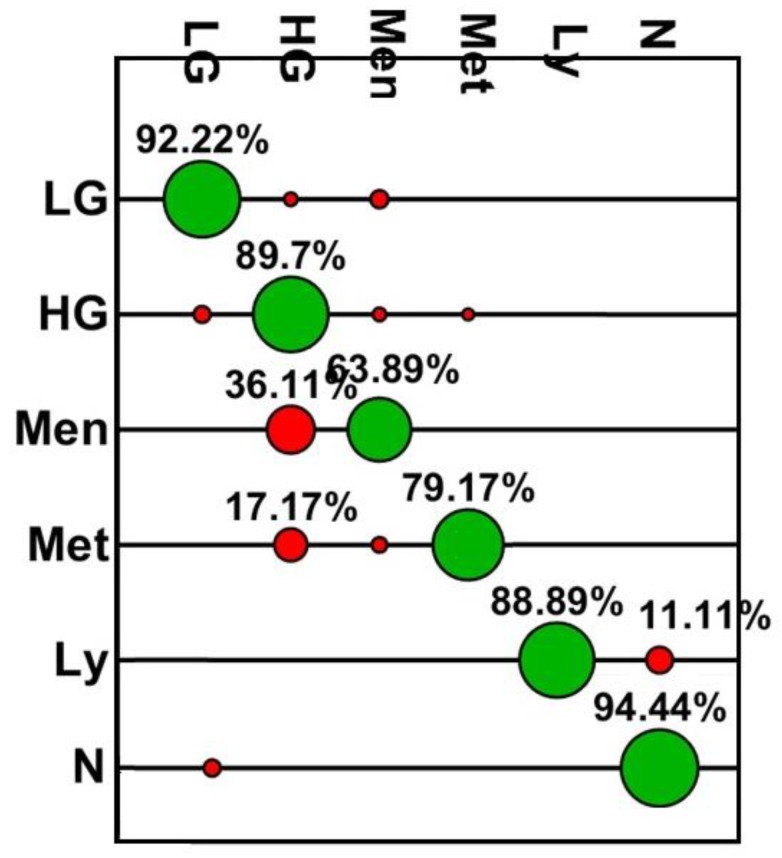
Graphical confusion matrix for PCA-LDC model using smear-based results. Key: N; Non tumour brain tissue, LG; Low-grade Glioma, HG; High-grade Glioma, Men; Meningioma, Met: Metastasis, Ly; Lymphoma. Green demonstrates those correctly classified, whereas red indicates an incorrect classification.

**Figure 4:**
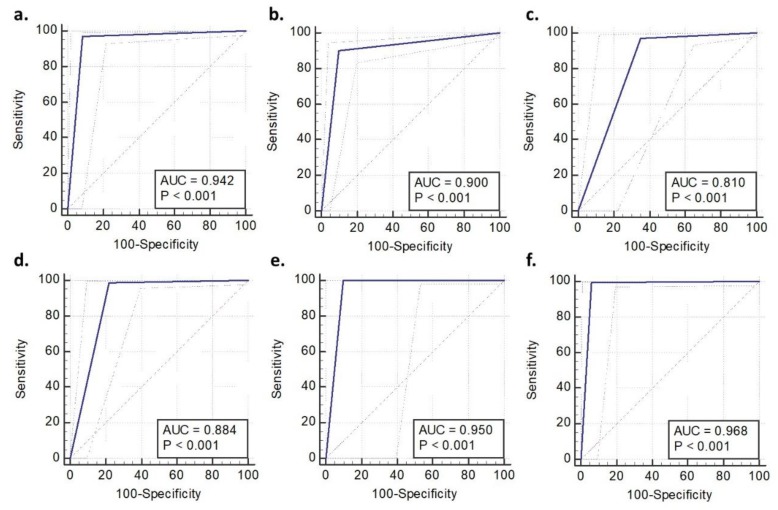
Receiver operating characteristic curves for smear-based samples: (**a**) Low-grade Glioma; (**b**) High-grade Glioma; (**c**) Meningioma; (**d**) Metastasis; (**e**) Lymphoma; and, (**f**) Non tumour brain tissue. Dashed pale blue curves represent 95% confidence intervals (AUC: area under the curve).

From Figure 3 it can be seen that the accuracy for detection of primary brain tumours was between 64% and 92%. The algorithm provided the lowest accuracy for meningioma (64%) with differentiation of glial tumours proving more robust (92.2 and 89.7%). The ROC parameters and curves (Figure 4, Table 2) demonstrate the sensitivities and specificities range from 64%–94% and 91%–100%, respectively, again with meningioma falling behind the other tumours for sensitivity. As the area under the curve is >0.8 for all tumour classifications it confirms the high accuracy of the classification model and presence of statistical significance (*P* < 0.001). This is an important result if this model is to provide clinically useful information. With the exception of meningioma the positive and negative predictive values are consistently high (Table 2), with all negative predictive values over 95%.

### 3.2. SERS Results Compared to FFPE Tissue Results

**Figure 5:**
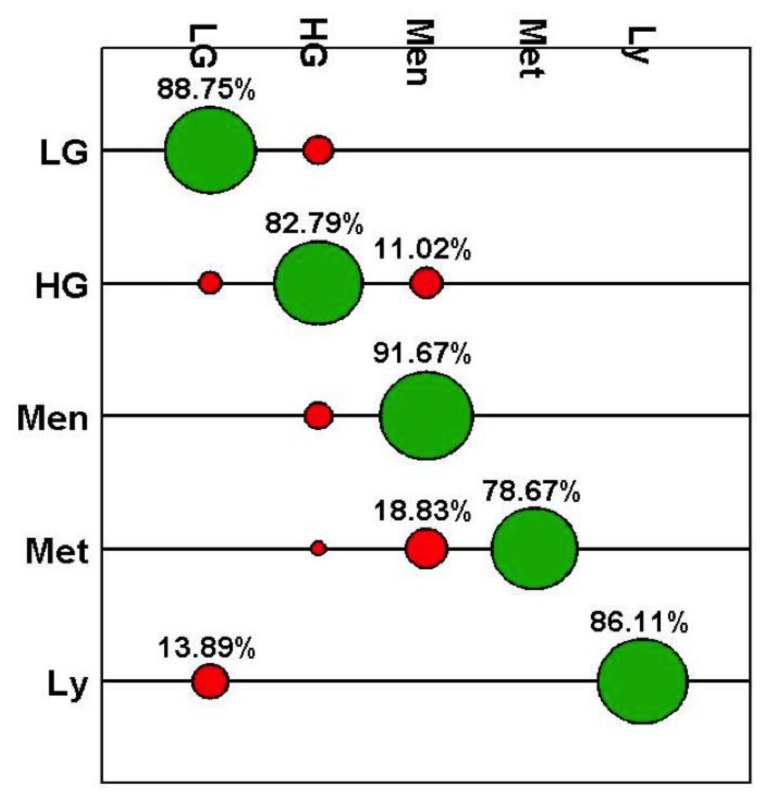
Graphical confusion matrix for PCA-LDC model using formalin fixed paraffin-embedded tissue results. Key: LG; Low-grade Glioma, HG; High-grade Glioma, Men; Meningioma, Met: Metastasis, Ly; Lymphoma. Green demonstrates those correctly classified, whereas red indicates an incorrect classification.

**Figure 6:**
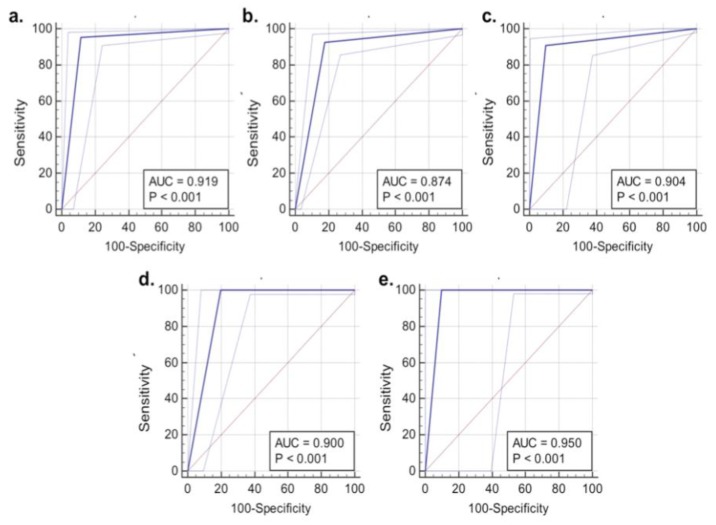
Receiver operating characteristic curves for formalin-fixed paraffin-embedded tissue results: (**a**) Low-grade Glioma; (**b**) High-grade Glioma; (**c**) Meningioma; (**d**) Metastasis; (**e**) Lymphoma. Dashed pale blue curves represent 95% confidence intervals (AUC: area under the curve).

When comparing the SERS results to the final FFPE diagnosis, the classification model also works with a high degree of accuracy. With the exception of metastatic tumours, the accuracy dips slightly for all cases as compared to the smear results (Figure 5, Table 3). This may be due to a variety of reasons, including non-tumour brain tissue within the biopsy material or areas of necrosis. Given this is not possible to determine macroscopically by eye, this remains a limitation of the study. The reduction in classification accuracy is to be expected as the neuropathologist has many diagnostic tools to aid the final FFPE diagnosis such as tumour morphology, architecture and immunohistochemical testing. The ROC graphs though do continue to show the reliability and statistical significance of the classification model (Figure 6), highlighting the ability of SERS to differentiate the tumour types within this study. Following formalin fixation all samples were found to contain tumour tissue therefore no non-tumour samples are represented within this analysis.

## 4. Discussion

Many Raman spectroscopic studies have been performed in recent years with the aim of introducing a clinically useful diagnostic tool that is easy to use and reagent-free. Much work has been performed towards standardisation of methodology and analysis, as this has previously led to criticism as many different techniques have been used [24]. Previous work within the field has shown good discrimination between normal and cancerous tissue. For example, within brain tumours, prostate and ovarian cancer we have previously found potential using Raman spectroscopy to differentiate normal from tumour within both tissue and biofluids [5,25,26,27]. The aim of this study was to determine if a handheld Raman probe could provide comparable results to both an intraoperative smear preparation and the final FFPE histological diagnosis. Comparable results would allow for further exploration of a Raman based probe for intraoperative use, particularly within the field of neuro-oncology. The use of fresh tissue, within a neuropathology laboratory, testing samples sent for smear preparations demonstrates a novel approach within this field, moving spectroscopic assessment closer to the patient. This study was designed to be a snap-shot of a typical clinical setting in a neuro-oncology surgical department over a 6-month period in order to ascertain the potential of extending our investigations into the potential translatability of this approach for routine practice.

These results demonstrate the ability of a handheld Raman device, when combined with gold nanoparticles, to differentiate tumour types from fresh brain tissue. The results are comparable to both the intraoperative smear preparations and final FFPE diagnosis, with accuracy at detecting a variety of primary brain tumours and metastases ranging from 63.9–94.4% as compared to the intraoperative smear preparation, and 78.7–91.7% when compared to the FFPE diagnosis. With the exception of meningioma the sensitivities and specificities are above 75% throughout, with the majority over 90%. The PPV and NPV results are also consistently high. It is possible that the meningioma group demonstrated lower accuracies due to varying morphological appearances. A much larger study would be required to determine differences between meningiomas of different types and grade. A similar issue applies to metastasis. This is a difficult group to combine as they are from different primary sites and therefore a different phenotype which will be expressed and would likely account for the differences in spectral classification. Due to the small number of metastatic tumours it was not possible to sub-classify these based on spectra within this study. This would be a next step when taking this study forward into a larger test group. These results are also comparable to a recent study demonstrating the possible use of Raman to detect tumours prior to biopsy [14]. For a test to be clinically useful, especially intraoperatively, a high accuracy, PPV and NPV is needed. These results compare well to a study performed on intraoperative smears and the final results compared to the FFPE diagnosis, which yielded an accuracy of 95.25% with PPV of 95.3% and NPV of 95.1% [28]. This is an important step as it allows the results to be comparative to current techniques, possibly demonstrating an improvement. By adequately training the Raman probe these results demonstrate a possible improvement on the current method of intraoperative smear diagnosis, reducing the human element involved and decreasing time to reach a diagnosis. As the accuracy of the Raman probe is slightly reduced when results are compared to the FFPE diagnosis for the majority of tumours (see Figure 3 and Figure 5), the role for conventional neuropathology remains, with this tool focused towards intraoperative diagnosis. As this technique is taken forward, it would also require improvements within the Raman spectrometer to reduce the signal to noise ratio, in order for the nanoparticles to no longer be required, as one of the leading benefits to spectroscopy is the lack of labelling required. They were however felt to be important to this study given it is an early step in the introduction of spectroscopy into a surgical theatre. 

These positive findings indicate the possible benefits to having a handheld Raman device present within the neurosurgical theatre, although much larger datasets need to be explored before clinical trial, in particular for classes that had a small number of samples in this study, such as lymphoma and metastasis. As all tissue was preserved following spectral acquisition and fixed to aid final diagnosis, we have also shown that spectral acquisition and addition of nanoparticles have not harmed the tissue, nor prevented final histological diagnosis as a final diagnosis by a Consultant Neuropathologist was possible in all cases. This is an important step when bringing this technology into the clinical field. Patient factors were not considered within this study as they were felt unlikely to directly influence the histopathological assessment. As the technique is developed, it may prove useful to add patient characteristics into an algorithm to improve accuracy, particularly within the paediatric field as some tumours are inherent to certain age groups.

When comparing these results to the final FFPE, it is understood that a final histopathological diagnosis includes many factors, including molecular analysis. The comparison was performed within this study to demonstrate differences primarily between an intraoperative result and the FFPE. Further studies would be required to determine if the Raman spectral differences were able to differentiate the underlying molecular changes, thus circumventing the need for molecular testing. 

These results would suggest a handheld device within theatres, may be able to assist surgeons in removing tumour tissue without the need for an intraoperative smear preparation. This could reduce surgical time as no result is awaited and allow for improved surgical resection as small foci of tumour could be identified. It can be seen that the time taken to prepare and take the spectra is similar to that required to produce a smear preparation, therefore the time saved is within the analysis and removal of the need to send the sample to the pathology laboratory. Moving this study forward, it would be important to study the junction between non-tumour brain tissue, brain tissue infiltrated by tumour cells and tumour tissue to understand the threshold at which the Raman probe is able to detect tumour cells. This would therefore allow demonstration of any benefit of its use over current methods.

As the classification model is able to determine tumour type this also would allow for further management steps to be completed, such as the addition of Gliadel wafers in the case of high-grade gliomas. The use of intracranial chemotherapy, such as Gliadel, is recommended by the National Institute for Clinical Excellence (NICE) under certain conditions, one of which is the diagnosis intraoperatively of a high-grade glioma by a neuropathologist [29]. Raman spectroscopy could therefore be used to circumvent the need to involve the neuropathologist, streamlining processes within theatre. The identification of a metastatic tumour is also important when planning the level of resection undertaken. We have not used the results to determine primary tumour origin for metastatic tumours, as this has previously been shown to be challenging, particularly for cases such as adenocarcinomas from different primary sites [30]. Intraoperatively, the determination of a metastasis versus a primary brain tumour is the level required and offered from an intraoperative smear preparation. Therefore allowing conventional histopathology and immunohistochemistry to determine the primary site of origin is the most logical step.

Determination of surgical margins within breast cancer has been demonstrating using Raman spectroscopy [19]. If developed, our classification model may also allow for other surgical sites to determine presence of absence of tumour intraoperatively, again removing the need for intraoperative frozen sections to be performed and improve resection clearance. Additional information such as samples descriptive statistics (e.g., age, gender and tumour location) would benefit the interpretation of the analysis results to provide a more patient-driven diagnosis. Herein, these factors were not considered, which limits this paper to the specific features of the cohort analysed. Nevertheless, Raman spectroscopy combined with chemometric algorithms has shown great potential for tumour differentiation, evidenced on the high accuracies, sensitivities and specificities achieved for this data set.

## 5. Conclusions

Overall, this study presents a novel approach to intraoperative brain tumour diagnosis and is one of the first studies to report results on intraoperative fresh brain tumour samples. The next step is to move this technology into theatre and continue to develop the classification model to allow for real-time feedback to the surgeon and allow Raman technology to reach its full potential.

## Figures and Tables

**Table 1:** Results of both intraoperative smear preparations and final formalin-fixed paraffin-embedded tissue for each case tested.

Case Number	Smear Result	Paraffin Result
1	Low-grade glioma	Glioblastoma
2	Meningioma	Meningioma
3	Metastasis	Ovarian serous carcinoma
4	High-grade glioma	Glioblastoma
5	High-grade glioma	Glioblastoma
6	Meningioma	Meningioma
7	Metastasis	Adenocarcinoma
8	High-grade glioma	Glioblastoma
9	High-grade glioma	Glioblastoma
10	Metastasis	Renal cell carcinoma
11	Metastasis	Lung adenocarcinoma
12	no tumour	Glioblastoma
13	Low-grade glioma	Astrocytoma Grade 2
14	Inflammation	Astrocytoma Grade 2
15	Inflammation	Astrocytoma Grade 2
16	Metastasis	Ovarian serous carcinoma
17	High-grade glioma	Glioblastoma
18	High-grade glioma	Glioblastoma
19	High-grade glioma	Glioblastoma
20	High-grade glioma	Glioblastoma
21	High-grade glioma	Glioblastoma
22	reactive Low-grade glioma	Low grade glioma
23	Intermediate-grade glioma	Glioblastoma
24	Low-grade glioma	Astrocytoma Grade 3
25	Lymphoma	High grade B cell lymphoma
26	Glioma	Astrocytoma Grade 2
27	No definite tumour	Astrocytoma Grade 2
28	Low- to intermediate-grade glioma	Astrocytoma Grade 2
29	High-grade glioma	Glioblastoma

**Table 2:** Figures of merit for PCA-LDC model using smear-based samples. Cohen’s kappa coefficient (κ) = 0.87.

Class	Accuracy (%)	Sensitivity (%)	Specificity (%)	PPV (%)	NPV (%)
N	98.6	94.4	99.5	97.7	98.8
LG	96.1	92.2	97.0	88.7	98.0
HG	90.3	89.7	90.6	83.5	94.4
Men	94.8	63.9	97.1	62.1	97.3
Met	95.4	79.2	98.8	93.3	95.8
Lv	99.6	88.9	100	100	99.6

Key: N; Non-tumour brain tissue, LG; Low-grade Glioma, HG; High-grade Glioma, Men; Meningioma, Met: Metastasis, Ly; Lymphoma, PPV; positive predictive value, NPV; negative predictive value.

**Table 3:** Figures of merit for PCA-LDC model using paraffin-embedded tissue results. Cohen’s kappa coefficient (κ) = 0.85.

Class	Accuracy (%)	Sensitivity (%)	Specificity (%)	PPV (%)	NPV (%)
LG	93.8	88.7	95.4	85.8	96.4
HG	88.0	82.8	92.8	91.6	85.1
Men	90.8	91.7	90.8	42.4	99.3
Met	96.3	78.7	100	100	95.7
Lv	99.5	86.1	100	100	99.5

Key: LG; Low-grade Glioma, HG; High-grade Glioma, Men; Meningioma, Met: Metastasis, Ly; Lymphoma, PPV; positive predictive value, NPV; negative predictive value.
